# Structural and functional comparison of magnesium transporters throughout evolution

**DOI:** 10.1007/s00018-022-04442-8

**Published:** 2022-07-12

**Authors:** G. A. C. Franken, M. A. Huynen, L. A. Martínez-Cruz, R. J. M. Bindels, J. H. F. de Baaij

**Affiliations:** 1 Department of Physiology, Radboud Institute for Molecular Life Sciences, Radboud University Medical Center, P.O. Box 9101, 6500 HB Nijmegen, The Netherlands; 2 Center for Molecular and Biomolecular Informatics, Radboud Institute for Molecular Life Sciences, Radboud University Medical Center, Nijmegen, The Netherlands; 3 Center for Cooperative Research in Biosciences (CIC bioGUNE), Bizkaia Science and Technology Park, Derio, 48160 Bizkaia Spain

**Keywords:** Magnesium, Channel, Transporter, CNNM, TRPM, SLC41

## Abstract

Magnesium (Mg^2+^) is the most prevalent divalent intracellular cation. As co-factor in many enzymatic reactions, Mg^2+^ is essential for protein synthesis, energy production, and DNA stability. Disturbances in intracellular Mg^2+^ concentrations, therefore, unequivocally result in delayed cell growth and metabolic defects. To maintain physiological Mg^2+^ levels, all organisms rely on balanced Mg^2+^ influx and efflux via Mg^2+^ channels and transporters. This review compares the structure and the function of prokaryotic Mg^2+^ transporters and their eukaryotic counterparts. In prokaryotes, cellular Mg^2+^ homeostasis is orchestrated via the CorA, MgtA/B, MgtE, and CorB/C Mg^2+^ transporters. For CorA, MgtE, and CorB/C, the motifs that form the selectivity pore are conserved during evolution. These findings suggest that CNNM proteins, the vertebrate orthologues of CorB/C, also have Mg^2+^ transport capacity. Whereas CorA and CorB/C proteins share the gross quaternary structure and functional properties with their respective orthologues, the MgtE channel only shares the selectivity pore with SLC41 Na^+^/Mg^2+^ transporters. In eukaryotes, TRPM6 and TRPM7 Mg^2+^ channels provide an additional Mg^2+^ transport mechanism, consisting of a fusion of channel with a kinase. The unique features these TRP channels allow the integration of hormonal, cellular, and transcriptional regulatory pathways that determine their Mg^2+^ transport capacity. Our review demonstrates that understanding the structure and function of prokaryotic magnesiotropic proteins aids in our basic understanding of Mg^2+^ transport.

## Introduction

Magnesium (Mg^2+^) is required as co-factor in over 300 enzymatic reactions and is therefore involved in many physiological processes [1–3]. The involvement of free Mg^2+^ can be via substrate complexes or directly to the enzymes themselves and is dependent on the spatial arrangement of water molecules [2]. This is influenced by the large hydration shell, which is 400 times larger when unhydrated and larger than other positively charged minerals, such as Na^+^, K^+^ and Ca^2+^ [4]. In pro- and eukaryotic cells, the majority (± 90%) of the intracellular Mg^2+^ is bound to ATP (MgATP). Among others, MgATP is essential for ATPase function, phosphorylation events, and glycolytic enzymes [5–8]. Ionised Mg^2+^ acts as a co-factor for enzymes important for macromolecule synthesis, such as DNA/RNA polymerases and tRNA synthetases [9–11]. Moreover, Mg^2+^ plays a central role in protein synthesis. Data from *E.coli* bacteria indicate that a single ribosome contains at least 170 Mg^2+^ ions [12]. In photosynthesis, Mg^2+^ is located in chlorophyll molecules and crucial for the absorption of photons that is required for ATP and O_2_ production, a phenomenon that supports all multicellular organisms [13]. Moreover, Mg^2+^ is an antagonist for Ca^2+^, which is of particular importance in the regulation of ion channel activity [1].

**Fig. 1:**
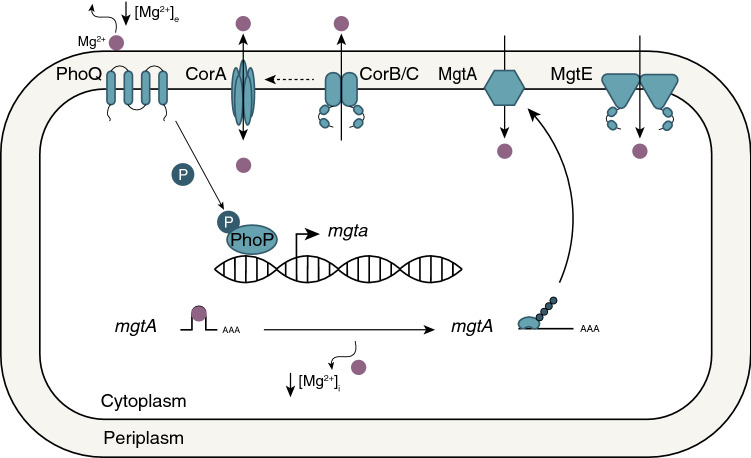
Schematic overview of prokaryotic Mg^2+^ transport proteins. Cobalt resistance (Cor) and Mg^2+^ transporting (Mgt) proteins CorA or MgtE form the major Mg^2+^ influx systems in prokaryotes. However, the channels are rarely present together in the same species, i.e. prokaryotes either have CorA or MgtE channels. CorA and CorB/C can regulate Mg^2+^ efflux from cells, although the dependency of CorA in relationship to CorB/C proteins remains unstudied. The MgtA ATPase is activated when extra- or intracellular Mg^2+^ levels are low. In response to these cues, the PhoQ/P system is activated. Upon Mg^2+^ restriction, PhoQ phosphorylates PhoP, which in turn results in transcription of *mgta* encoding MgtA. Low intracellular Mg^2+^ concentration also enables efficient translation of the *mgta* transcript via a riboswitch. Translation results in expression of the MgtA ATPase, which hereafter localises to the membrane to regulate Mg^2+^ influx via primary active transport. The intracellular Mg^2+^ concentration is ultimately determined based on the expression of the channels/transporters at the membrane and their activity

As Mg^2+^ is central to enzymatic function and metabolism, cells require a transport system to keep Mg^2+^ levels stable. In vertebrates, the main magnesium-transporting proteins are transient receptor potential melastatin (TRPM) 6 and -7, solute carrier 41 (SLC41), cyclin M (CNNM) proteins, and mitochondrial RNA splicing protein 2 (Mrs2) (Table 1). These Mg^2+^ channels and transporters often have a prokaryotic, bacterial and/or archaeal, orthologue. Although their function is to facilitate Mg^2+^ fluxes, they are also permeable for other (trace) divalent cations (Table 2). While research generally focusses on characterisation of eukaryotic Mg^2+^ transporters in mammalian cell models, many valuable insights can be obtained by examining their prokaryotic counterparts in greater detail. In prokaryotes, four major Mg^2+^ channels and transporters have been identified, named after their role in Cobalt resistance (Cor) and Mg^2+^ transport (Mgt): CorA, CorB/C, MgtA/B, and MgtE (Fig. 1). In recent years, structures of several prokaryotic Mg^2^^+^ transporters and channels have been elucidated using cryo-electron microscopy and X-ray crystallography. Not only have these structures given insights into how these transporters/channels are regulated, but also reveal the function of their eukaryotic counterparts.

**Table 1:** Overview of proteins found in prokaryotes that regulate cellular Mg^2+^ levels and their orthologue families in eukaryotes

Prokaryote	Eukaryote		
	*S. cerevisae*	*Plantae*	*Metazoa*
Superfamilies			
CorA	Mrs2, Alr1/2, Mnr2, Lpe10	Mrs2-like proteins	Mrs2
MgtA	–	–	–
MgtE	–	MgtE-like proteins^a^	SLC41
CorB/C	MAM3	DUF21(-CBS) proteins	CNNMs
–	–^b^	–	TRPM6/7

^a^The MgtE orthologues have currently only been described in unicellular green and red algae (*Viridiplantae and Archaeplastida*, respectively) [14, 15]

^b^TRP channels have been described in yeast, but to date no particular orthologues of TRPM6/7 have been identified

**Table 2:** Overview of prokaryotic and eukaryotic Mg^2+^ channels and transporters and their ion selectivity

Protein	Transporting mechanism	Ion selectivity	Technique [reference]
CorA	Channel	Ca^2+^ > Mn^2+^ > Co^2+^ > Mg^2+^ > Ni^2+^ (in the constitutively open CorA D253K mutant)	Voltage clamp recording in oocytes [16]
		Mg^2+^ > Mn^2+^ > Co^2+^ > Ni^2+^ > Ca^2+^	Competition assay in *S. typhimurium* [17]
Mrs2	Channel	Mg^2+^ > Ni^2+^ > Ca^2+^ = Mn^2+^ = Co^2+^	Patch clamp recording in yeast [18]
CorB/C	Exchanger	Not reported	
CNNM2	Transporter (?)	Mg^2+^ > Sr^2+^ = Zn^2+^ = Cd^2+^ = Ni^2+^ > Ba^2+^ = Co^2+^ > Fe^2+^ = Cu^2+^ = Mn^2+^ = Ca^2+^	Voltage clamp technique in oocytes [19]
CNNM3	Exchanger (?)	Mg^2+^ > Fe^2+^ > Cu^2+^ > Co^2+^ > Ni^2+^ > Ca^2+^	Voltage clamp technique in oocytes [19]
MgtE	Channel	Mg^2+^ > Mn^2+^ > Ca^2+^ > Na^+^ = K^+^	Liposome-based transport assays [20]
SLC41A1	Na^+^-exchanger (?)	Mg^2+^ > Sr^2+^ = Fe^2+^ ≥ Ba^2+^ = Cu^2+^ > Zn^2+^ = Co^2+^ > Cd^2+^ = Mn^2+^ = Ni^2+^ = Ca^2+^	Voltage clamp technique in oocytes [19]
SLC41A2	Na^+^-exchanger (?)	Mg^2+^ > Ba^2+^ > Ni^2+^ = Co^2+^ > Fe^2+^ = Mn^2+^ = Sr^2+^ > Cu^2+^ = Zn^2+^ = Ca^2+^	Voltage clamp technique in oocytes [19]
SLC41A3	Na^+^-exchanger (?)	Ba^2+^ > Mg^2+^ > Ni^2+^ = Zn^2+^ > Sr^2+^ = Fe^2+^ > Mn^2+^ > Cu^2+^ = Co^2+^ > Ca^2+^	Voltage clamp technique in oocytes [19]
TRPM6	Channel	Zn^2+^ > Ba^2+^ > Mg^2+^ = Ca^2+^ = Mn^2+^ > Sr^2+^ > Cd^2+^ = Ni^2+^	Patch clamp recording in CHOK1 cells [21]
		Ba^2+^ > Ni^2+^ > Mg^2+^ > Zn^2+^ ≥ Ca^2+^	Patch clamp recording in HEK293 cells [22]
TRPM7	Channel	Zn^2+^ = Ni^2+^ > Ba^2+^ > Co^2+^ > Mg^2+^ ≥ Mn^2+^ ≥ Sr^2+^ ≥ Cd^2+^ ≥ Ca^2+^	Patch clamp recording in HEK293 cells [23]
		Ni^2+^ > Zn^2+^ > Ba^2+^ = Mg^2+^ > Ca^2+^ = Mn^2+^ = Sr^2+^ > Cd^2+^	Patch clamp recording in CHOK1 cells [21]
MgtA	P-type Mg^2+^-ATPase	Zn^2+^ > Mg^2+^ > Ni^2+^ = Co^2+^ > Ca^2+^	Competition assay in *S. typhimurium* [17]
MgtB	P-type Mg^2+^-ATPase	Mg^2+^ = Co^2+^ = Ni^2+^ > Mn^2+^ > Ca^2+^	Competition assay in *S. typhimurium* [17]

To note, two-electrode voltage clamp can only be used in relatively large in vitro models, e.g. oocytes. In addition, the intracellular compartment cannot be controlled and may therefore be not suitable to determine permeation profiles [24, 25]

In this review, we compare the structure of the prokaryotic Mg^2+^ transporting proteins and interpret the functional similarities of their eukaryotic orthologues. All Mg^2+^ transporting superfamilies will be discussed in terms of structure and functional characteristics.

## Main body

### The CorA family and the mitochondrial Mg^2+^ channel Mrs2 orthologue

In 1969, two groups identified active Mg^2+^ transport in *E. coli*, which was temperature dependent, but independent of other cations, such as calcium (Ca^2+^), potassium (K^+^), or manganese (Mn^2+^) [26, 27]. The molecular mechanism for Mg^2+^ transport was identified in the context of cobalt (Co^2+^) resistance. Exposure of *E.coli* to relatively high Co^2+^ levels disrupted growth, yet was inhibited in the presence of high Mg^2+^ levels [28]. Mutants that displayed resistance to Co^2+^-mediated growth retardation also showed decreased Mg^2+^ transport, suggestive for shared uptake of these metals into bacteria [28]. The gene was identified in *Salmonella typhimurium* and named *corA* (protein: CorA) [29]. Approximately half of the prokaryotes have the orthologue and is considered one of the main channels for Mg^2+^ into cells (Fig. 1) [30]. Transport studies using radioactive ^28^Mg^2+^ also showed that CorA also allows efflux, which is dependent on the extracellular Mg^2+^ concentration [31]. Efflux was abolished upon mutagenesis of the genetic loci encoding Cobalt resistance B, C, and D (CorB, -C, and -D; literature on the characterisation of CorD is absent and will not be described further) [31, 32]. Similarly to MgtE, CorA uses the electrochemical gradient across the cytoplasmic membrane to transport its substrates [33, 34]. This dependence on the membrane potential means that the ion transport it promotes is influenced by changes in pH or by fluctuations in the concentration of other ions. CorA is, together with MgtE, the only primary Mg^2+^ channel whose crystal structure is known in its entirety in the presence and in the absence of divalent cations (Mg^2+^ and Ca^2+^) [35–38].

**Fig. 2:**
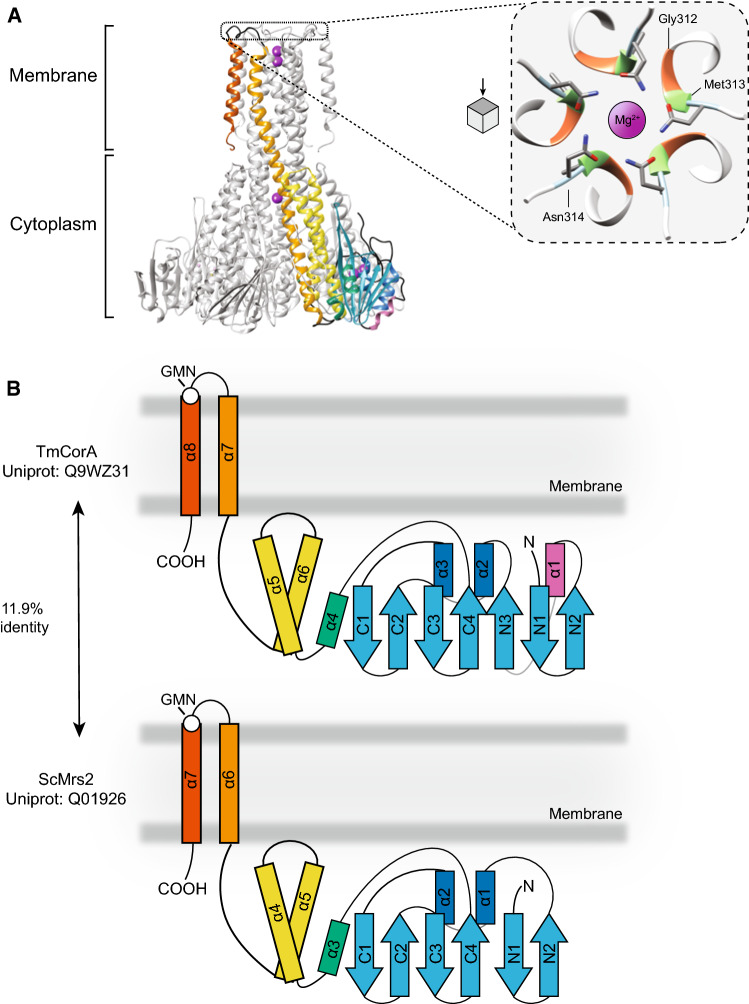
Structure of CorA and its orthologue Mrs2; **A** Structure of the pentamer Cobalt resistance A of *Thermotoga maritima* (TmCorA, PDB: 3JCF) in complex with Mg^2+^ (purple spheres, left panel) with one monomer highlighted. Right panel: zoom in on the surface of the transmembrane domain of CorA depicting the typical GxN motif that orthologues of CorA contain. Mg^2+^ ions have been enlarged for illustration purposes. **B** Schematic depiction of the monomer of CorA (up panel) and Mrs2 (bottom panel), with the location of the GMN motif located at the surface of the pore (white dot). Same colours as in A have been used to reflect the approximate strucuture and location. The schematic structure of the yeast homologue Mrs2 (PDB: 3RKG) has been depicted, which is based on on the cytoplasmic region

The structure of CorA was solved in the bacterium *Thermotoga maritima* (TmCorA) using X-ray crystallography [34, 39]. The protein consists of a large N-terminal region, connected to a smaller C-terminal region through a long α-helix. The C-terminal region contains two transmembrane helices. To be functionally active, CorA associates with itself to form funnel-shaped homopentamers, which in total contain ten transmembrane segments. The functional unit forms a central pore that crosses the membrane and reaches the intracellular region [35, 40]. The crystal structure revealed that cations bind to both the central pore and the intracellular region. The latter has regions rich in acidic residues that are located between the different subunits, where Mg^2+^ ions bind and regulate channel activity [34, 35, 40]. In each subunit there are five Mg^2+^-binding sites. Upon binding, Mg^2+^ ions increase the number of contacts between subunits and stabilise the closed conformation of the channel [38]. In the presence of Mg^2+^, the pore is too narrow to allow ion entry [41]. In contrast, loss of binding of Mg^2+^, the cytoplasmic N-terminus and gains flexibility, resulting in an asymmetric domain rearrangement. Ultimately, this allows the opening of the pore and influx of Mg^2+^ through the channel [42]. Yet, mutagenesis of the Mg^2+^-binding sites in TmCorA did not result in a constitutive opening of the channel, leaving the mechanistic role of Mg^2+^ in CorA gating unresolved [43]. Although the exact mechanism of opening or closing remains unknown, it has been postulated that the selectivity of CorA for Mg^2+^ is due to a conserved motif located at the entrance of the pore. This motif, defined by a YGMNFxxMPEL sequence, located at the loop of the C-terminal transmembrane alpha helices (Fig. 2) [44]. Distant orthologues of CorA lost the MPEL motif and only share the conservation of the Gly-X-Asn (GxN) motif, of which the X represents hydrophobic amino acids Met, Val, or Ile (Fig. 2) [44]. CorA is likely permeable for hexa-hydrated Mg^2+^, as it supports transport of Co^2+^ and Ni^2+^, which have the approximate same size as hydrated Mg^2+^ [17, 32]. In addition, CorA could be inhibited to cobalt hexamine, a structural analogue of Mg^2+^ as it competes with Mg^2+^-binding resides in the cytosolic pore domain [41].

During evolution, this pentameric transporter remained important for Mg^2+^ transport across all phyla (Table 1), as there are orthologues present in every phylum, as extensively described in this review [44]. The first eukaryotic orthologues of CorA were identified in *Saccharomyces cerevisiae*; mitochondrial RNA splicing protein 2 (Mrs2) and its homologue Lpe10. Both Mrs2 and Lpe10 are located in the mitochondria, possibly as a result of the endosymbiosis that gave rise to these organelles, and inactivating mutations in the genes cause decrease in Mg^2+^ content in mitochondria and cells [45–48]. The Mrs2 protein mainly shows structural conservation to CorA and has low amino acid identity to it (11.9%), apart from the typical GxN motif in the pore (Fig. 2B). The α–β–α sandwich at the N-terminus is similar for CorA and Mrs2, although the latter contains an extra α–β at the start of the protein [49]. Only a few residues are conserved between TmCorA and Mrs2 that are important for Mg^2+^ sensing [49]. Nevertheless, CorA expression partially alleviates the phenotype in *mrs2*-deficient yeast, highlighting the bacterial ancestry to this prokaryotic protein [46, 50]. Metazoa only contain one *Mrs2* homologue, which exclusively is localised to the mitochondria [47]. Indeed, Mg^2+^ plays a role in mitochondrial processes like the citric acid cycle, reactive oxygen species (ROS) production, and apoptosis [51, 52]. In contrast to metazoa, many Mrs2 orthologues are present in plants, such as in *Arabidopsis thaliana*, containing ten genes encoding orthologues of *Mrs2* (*ARAth;Mgt),* which may be due to independent gene duplications (TF328433) [53]. *Arabidopsis Mrs2* is able to complement, at least to an extent, the growth of *mrs2* mutant *S. cerevisiae* grown in Mg^2+^ deficient conditions [54]. It is still not understood why many plants have multiple orthologues of *Mrs2*, although this may be explained by the spatial specific expression pattern [55–57]. Moreover, deficiency of one of the genes often leads to growth retardation, indicating that these proteins are non-redundant [54, 58].

### The Mg^2+^ transporting ATPase MgtA/B and orthologues

Studies demonstrating Mg^2+^ influx in bacteria demonstrated that the kinetics of Mg^2+^ transport changed based on the exposure to different extracellular Mg^2+^ concentrations [29]. Concentrations as low as 10 μmol/L were sufficient for bacterial growth and increased the Vmax of Mg^2+^ transport, suggesting there was more than one influx mechanism at hand. This observation ultimately led to the discovery of the *mgtA* and *mgtCB* loci in *S. typhimurium*, encoding for MgtA and MgtB/C, respectively [32].

The MgtA/B proteins belong to the P-type ATPase superfamily, which also includes the Na^+^/K^+^-ATPase and the Ca^2+^-ATPase, and use ATP hydrolysis to fuel Mg^2+^ transport [7]. *S. typhimurium* strains containing either wild-type MgtA or MgtB and mutant CorA displayed significant Mg^2+^ influx when exposed to 20 μmol/L Mg^2+^, with both MgtA and -B having a similar K_m_ as CorA in *S. typhimurium* [17]. Expression of the *mgtA* and *mgtCB* loci is modulated by the PhoQ/P two-component system, a phosphorylation relay that regulates virulence, pH, osmolality-induced stress, and Mg^2+^ deficiency (Fig. 1) [59–61]. The membrane receptor PhoQ phosphorylates the transcription factor PhoP when extracellular Mg^2+^ concentrations decrease. This initiates transcription of, among others, the *mgta* and *mgtCB* loci. In addition, the 5’ untranslated region (5’UTR) of *mgtA* undergoes conformational changes when intracellular Mg^2+^ levels are low as consequence of the release of Mg^2+^ ions of mRNA molecule and initiation of translation, a phenomenon known as riboswitch [62, 63]. This has led to the general belief that Mg^2+^ influx is mainly regulated by CorA, but is promoted by MgtA/B upon Mg^2+^ deprivation.

**Fig. 3:**
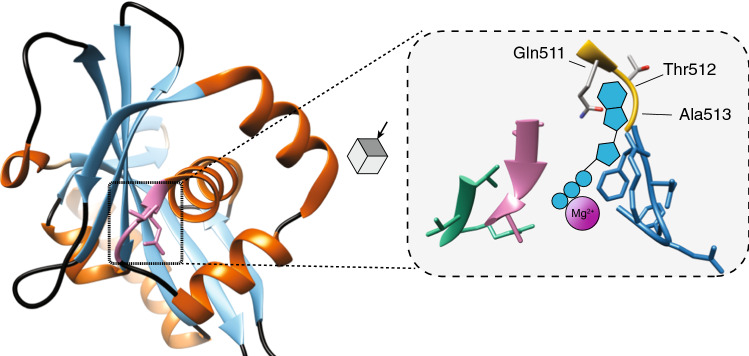
Structure of the N-terminus of MgtA. Structure of the N-terminus of Magnesium Transporter A (MgtA) of *Escherichia coli* (MgtA, PDB: 3GWI). Right panel: zoom in on the surface of the MgATP-binding site with the four binding motifs. The xTG (yellow) is unique to the MgtA protein compared to members of the P-type ATPases. MgATP has been enlarged for illustration purposes and does not reflect the physical bindings sites with the protein

Elucidation of the structure of the N-terminus of MgtA revealed the X-Thr-Gly motif (xTG), with X coding for Asn, Asp, Gln, or Glu, which is likely involved in binding of the MgATP (Fig. 3) [64]. This motif is one of the four ATP-binding motifs in MgtA and is unique compared to Ca^2+^ and Na^+^/K^+^-ATPases and shared in many of the MgtA homologues in various prokaryotes. In MgtB, the Thr is replaced by Ser in *S. typhimurium* (QSG) [64]. It has been postulated that QSG could result in higher affinity for the MgATP nucleotide base compared to the xTG motif, but this has not been experimentally validated yet. Further characterisation of the MgtA/B protein structure is needed to understand the role of its unique motif in of Mg^2+^ transport.

Although Mg^2^^+^-ATPases have been postulated in vertebrates, the molecular identity of a MgtA/B orthologue remains obscure. Many studies reported Mg^2+^-dependent ATP hydrolytic activity in different organelles, such as the plasma membrane, endoplasmatic reticulum, sarcolemma, and microvesicles found in heart, muscle, and brain [65–70]. This may suggest the presence of a Mg^2^^+^-ATPase, yet studies focussing on Mg^2+^ transport by these Mg^2+^-dependent ATPases are limited. Searching for orthologues of MgtA, homology detection using HHpred suggested that members of the ATPase 13 in human could be an interesting candidate [71, 72]. Inactivation of the *ATP13A4* gene was associated with delayed language development and in overexpression in cells stimulates Ca^2+^ influx [73, 74]. Members of the ATP13 family transport a range of electrolytes or organic compounds, such as Ca^2+^, Mn^2+^, or polyamines [75–77]. Interestingly, Claudin 16 knock-out mice, a model that induces renal-mediated Mg^2+^ wasting, showed increased gene expression of *Atp13a4* in the kidney [78]. In C. elegans, the orthologue of human ATP13A2, CATP-6, was found to regulate GON-2 [79]. GON-2 is the orthologue of the Mg^2+^ channel TRPM6 and -7 in vertebrates, proteins that will be discussed in detail later. Functional assays, preferably with purified ATP13A4 protein, could reveal whether these proteins could transport Mg^2+^ and are the orthologues of MgtA/B.

### The cellular Mg^2+^ channel MgtE and its vertebral orthologue SLC41

In a *corA*, *mgtA*, and *mgtCB* deficient *S. typhimurium* MM281 strain, a genomic library was expressed of the Gram-positive *Bacillus firmus* to identify additional Mg^2+^ transporters [80]. Cells that showed growth under Mg^2+^ deprived conditions were selected for further genetic analysis, which led to the discovery of the *mgte* locus [80]. The MgtE Mg^2+^ channel is present in both *Bacteria* and *Archaea*, although it appears to be largely absent in prokaryotes that express CorA (Fig. 1) [81]. Interestingly, the Gram-negative bacteria *Dechloromonas aromatica* and *Magnetospirilllum magnetotacticum* contain CorA homologues that are unusually long and have a N-terminus that exhibits homology to MgtE [44]. Just as CorA, MgtE is a non-selective cation channel, facilitating influx of Mg^2+^, Zn^2+^,Co^2+^, and Ni^2+^ [80]. Similar to *mgtA,* the *mgtE* transcript undergoes structural changes via a riboswitch upon Mg^2+^ deprivation, controlled by a tertiary structure called the M-box [82]. However, the M-box is not present in every species that expresses MgtE. For instance, *Bacillus halodurans*, *B. subtilis, Clostridium acetobutylicum, Vibrio cholae,* and *Chormobacterium violcaceum* have the M-box upstream of *mgte*, whereas this is absent in the *mgte* transcripts in *S. aureus*, *Cornebacterium glutamicum*, *Mycobacterium bovis* and *T. maritima*.

**Fig. 4:**
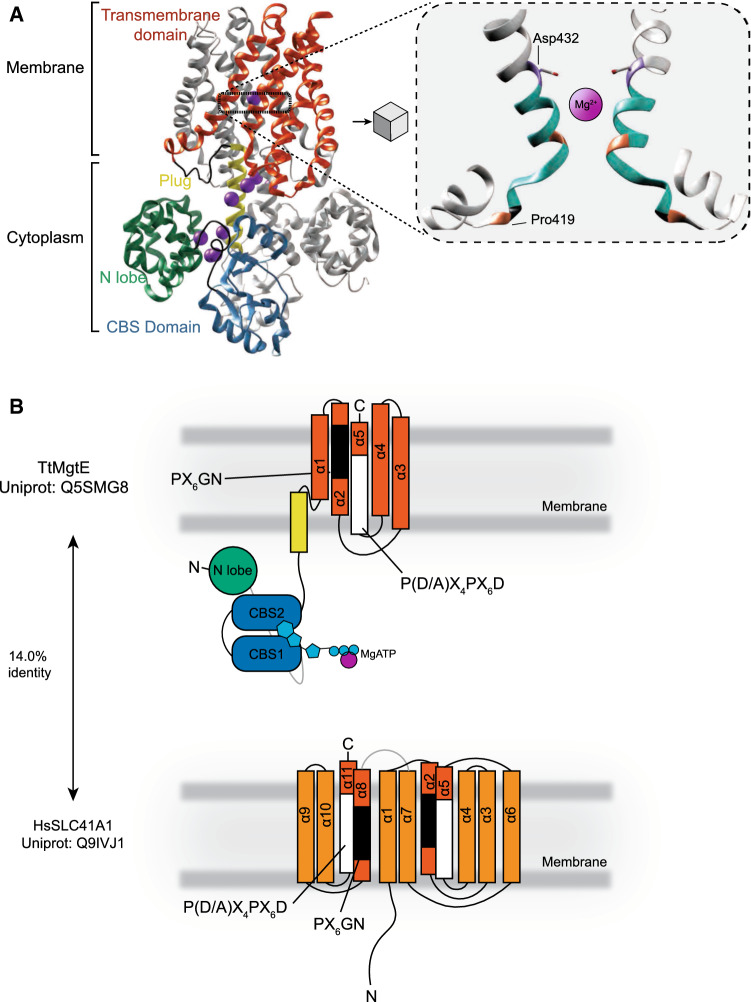
Structure of MgtE and its orthologue SLC41A1; **A** Structure of the dimer Thermus thermophilus Mg^2+^ transporter TtMgtE (PDB: 2ZY9) in complex with Mg^2+^ (purple spheres, left panel) with one monomer highlighted. Right panel: zoom in on the pore of MgtE depicting the typical P(D/A)X_4_PX_6_D motif that orthologues of MgtE contain. Both MgtE and solute carrier family 41 A 1 (SLC41A1) have the PX_6_GN and P(D/A)X_4_PX_6_D motifs. Mg^2+^ ions have been enlarged for illustration purposes. **B** Schematic depiction of the monomer of MgtE (upper panel) and SLC41A1 (bottom panel), with the approximate location of the PX_6_GN (black) and P(D/A)X_4_PX_6_D (white) domains. Same colours as in A have been used to reflect the approximate structure and location. The schematic structure of the human homologue SLC41A1 has been depicted, which has been based on the estimated structure using AlphaFold(AF-Q8IVJ1-F1)

MgtE adopts a homodimeric structure that differs structurally from the CorA proteins (Fig. 4A) [83]. The C-terminal tail contains cystathionine β-synthase (CBS) domains that are found in various proteins including chloride channels and AMP-activated protein kinase (AMPK). The CBS domains are heavily conserved and found in all phyla, with over 50 proteins in *H. sapiens* [84]. The domain in MgtE binds MgATP and has a dissociation constant (K_d_) for ATP of approximate 172 μmol/L, suggesting that MgATP is usually bound to the CBS domains as cytosolic ATP levels in vivo are in the millimolar range [85]. Additionally, Mg^2+^ ions bind to the N-lobe and plug (Fig. 4A), which is involved in the gating mechanism of the protein. Decreased intracellular Mg^2+^ levels give flexibility to the N-lobe and the plug, ultimately resulting in opening of the pore [20, 83, 86, 87]. The transmembrane spanning domains contain conserved D1 and D2 domains, defined by PX_6_GN and P(D/A)X_4_PX_6_D motifs, respectively. Located at helices TM2 and -5, these domains contribute to the specificity for cation transport of the MgtE proteins [81, 88, 89]. In MgtE, the N-lobe and plug contain Mg^2+^-bindings sites and are important for gating [20, 83, 86]. Through a strong interaction of the plug with the transmembrane, particularly TM2 and -5, the pore is closed. Loss of Mg^2+^ disrupts the association of the plug with TM2 and -5, ultimately leading to opening of the pore. The interaction of the N-lobe and CBS domains is ambiguous and disordered in Mg^2+^-free.

**Fig. 5:**
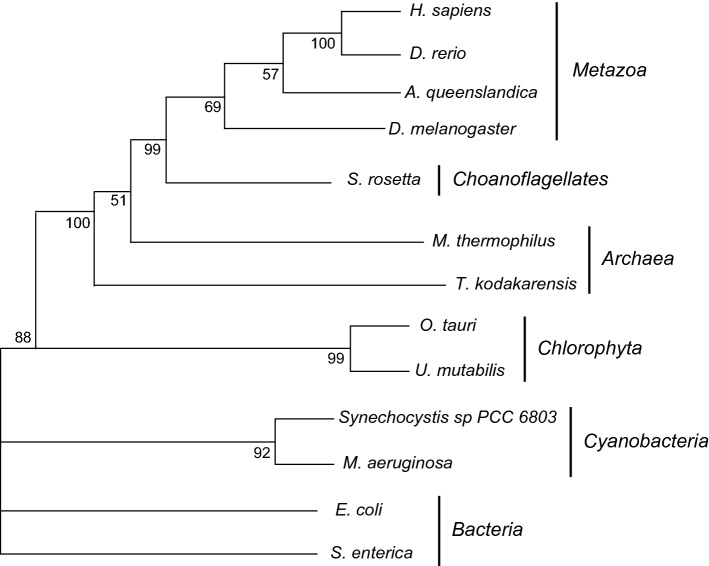
Phylogenetic tree of SLC41A1 orthologues. SLC41A1 orthologues are shared in all phyla, but limitedly in the *Plantae* kingdom. SLC41 orthologue sequences were searched with NCBI DELTA-BLAST, on Uniprot or ORCAE. Proteins sequences were then submitted to Pfam to confirm the presence of the conserved MgtE domain. Subsequently, a phylogenetic tree was constructed by maximum likelihood (bootstrap = 100) using MEGA11 [95]. Used sequences for SLC41A1 orthologues: *Homo sapiens:* NP_776253.3 (NCBI); *Danio rerio*: XP_002663867.1 (NCBI); *Drosophila melanogaster:* NP_001259335.1 (NCBI); *Amphimedon queenslandica*: P_003384010.3 (NCBI); *Salpingoeca rosetta*: XP_004993672.1 (NCBI); *Ostreococcus tauri*: XP_003084242.2 (NCBI); *Methanoculleus thermophilus*: SDK06600.1 (NCBI); *Synechocystis sp PCC 6803*: WP_010872029 (NCBI); *Ulva mutabilis:* UM021_0210.1 (ORCAE); Escherichia *coli*: A0A6M0PR42 (Uniprot). *Microcystis aeruginosa*: WP_052276493.1 (NCBI); *Thermococcus kodakarensis:* BAD85647.1 (NCBI); *Salmonella enterica:* A0A5U8SZT2 (NCBI). Branches were multifurcated when bootstrap values were < 50

In 2003, bioinformatical approaches led to the identification of the solute carrier family 41 (SLC41) in humans, mouse, and *C. elegans*, which is the eukaryotic homologue of MgtE [89]. Interestingly, MgtE orthologues have not been found in land plants, fungi and brown or red seaweed (Fig. 5) [15]. The identified proteins are homologous to the transmembrane spanning helices found in MgtE with the conserved motifs PX_6_GN and P(D/A)X_4_PX_6_D (Fig. 4B) [89]. The family has three members: SLC41A1, SLC41A2, and SLC41A3. The SLC41 family contains two times the PX_6_GN and P(D/A)X_4_PX_6_D motifs, allow to proteins to potentially function as monomers or dimers to facilitate Mg^2+^ transport, similarly to the prokaryotic MgtE proteins [90]. The double motifs are only present in *Archaea*, choanoflagellates, and metazoa, while uni- and multicellular algae and (cyano)bacteria share contain only one PX_6_GN and P(D/A)X_4_PX_6_D motif. In addition, the MgtE orthologues in *Archaea* and metazoa do not contain the CBS domains and the structure of the SLC41 family deviates considerably from the MgtE proteins (Fig. 4). In addition, bacterial MgtE orthologues act as a Mg^2+^ channel, while SLC41 family members have been reported to work as Na^+^/Mg^2+^ antiporters [89–94]. Taken together, this suggests that archaeal and metazoan MgtE/SLC41 orthologues have taken a different evolutionary path. Detailed knowledge on the structure is absent, yet it is clear that the SLC41 family is distinct from MgtE proteins and might be differently regulated. To investigate this, large-scale comparative, genomic analyses coupled to experimental studies are required to search for orthologues in different phyla, which to date are limited. This could enable the field to study in depth the evolutionary relationship between SLC41 proteins and MgtE on a genomic level, while also offering opportunities for further biochemical and functional characterisation.

Electrophysiological studies in *Xenopus laevis* oocytes demonstrated Mg^2+^ elicited currents upon mouse SLC41A1 overexpression, yet other divalent cations, such as Zn^2+^, Fe^2+^ and Cu^2+^ were also transported [91]. Transformation with pUC18 human SLC41A1 plasmids in the *S. typhimurium* MM281 strain, which is deficient in *corA*, *mgtA*, and *mgtCB*, displayed superior growth in Mg^2+^ depleted conditions compared to the those with empty pUC18 plasmids [92]. Expression of SLC41A1 resulted in decreased intracellular Mg^2+^ using the fluorescent-sensitive probe Mag-Fura-2. Moreover, Mg^2+^ extrusion was abrogated upon Na^+^ removal, suggesting a Na^+^/Mg^2+^ exchange function to facilitate Mg^2+^ extrusion [93]. Also for SLC41A2 and -A3, Na^+^-dependent Mg^2+^ transport has been observed [94, 96], yet cation specificity may differ between family members [97]. In contrast, Arjona et al. observed both Na^+^-independent Mg^2+^ uptake and extrusion using the stable isotope ^25^Mg^2+^[98], leaving the molecular mode of action of the SLC41 members to be elucidated. However, studies in vivo observed a clear role for SLC41A1 and A3 in systemic Mg^2+^ homeostasis. Knock-down of *slc41a1* in zebrafish larvae decreased the Mg^2+^ content and induced a transcriptional response of genes involved in Mg^2+^ homeostasis [98]. *Slc41a3* expression was increased in kidneys of mice fed with low-Mg^2+^ diets and *Slc41a3*^−/−^ mice displayed hypomagnesaemia and increased intestinal Mg^2+^ absorption [99, 100]. Yet, how SLC41A3 contributes to Mg^2+^ homeostasis remains to be elucidated. While SLC41A1 and -A2 are located at the plasma membrane, SLC41A3 is predominantly found in the mitochondria [90, 94, 98]. To date, no causal link has been made between mitochondrial Mg^2+^ transport and systemic Mg^2+^ homeostasis.

### The Mg^2+^ efflux proteins CorB/C and orthologue CNNM proteins

**Fig. 6:**
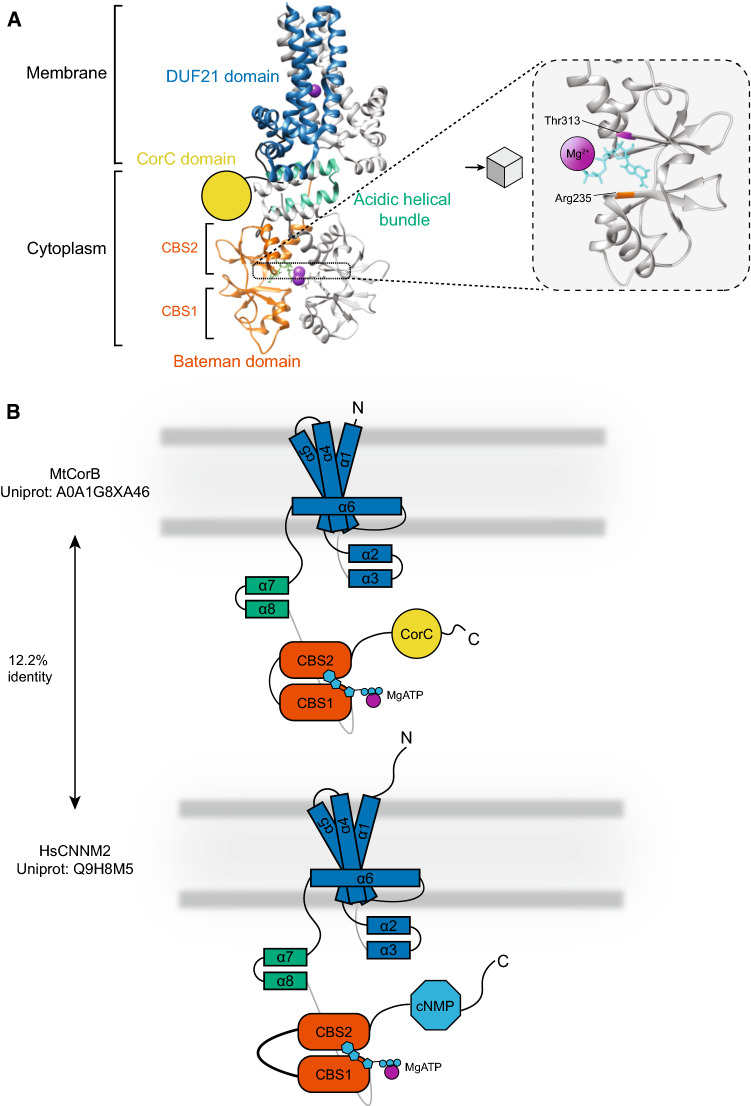
Structure of CorB and orthologue CNNM2; **A** Structure of the pentamer Methanoculleus thermophilus Cobalt of resistance CorB MtCorB (PDB: 7M1T) in complex with Mg^2+^-ATP (purple spheres, left panel) with one monomer highlighted. Right panel: zoom in on residues of the CBS domain that bind MgATP. Residues highlighted are homologues to human Cyclin M2 (hCNNM2) associated hypomagnesaemia, seizure, intellectual disability (HSMR) syndrome Thre568Ile (MtCorB-p.Thr313) and hCNNM4 associated Jalili syndrome Arg407Leu (Mt-CorB-p.Arg235). **B** Schematic depiction of the monomer of CorB (upper panel) and CNNM2 (bottom panel) using same colours as in A to reflect the approximate structure and location. The schematic structure of the human homologue has been depicted, which has been based on the estimated structure using homology modelling of CorB, as illustrated in Chen et al. (2021) [101]

Although CorA was initially thought to be involved in both Mg^2+^ influx and efflux, three other genes were identified in *S. typhimurium*; *corB*, *corC*, *corD* (Fig. 1)*.* These loci were initially identified in a screen for Co^2+^ resistance [31]. While CorA was essential for Mg^2+^ efflux, individual or combined inactivation of *corB*, *corC*, and *corD* disturbed efflux in bacteria that were preloaded with ^28^Mg^2+^ [31]. These three loci have a low level of identity with CorA, yet CorB and CorC display high similarity, with both containing CBS domains (Fig. 6). Functional and structural characterisation of CorD proteins have not been described to our knowledge, so its role in Mg^2+^ transport remains unknown. The pore of the protein is located in the domain of unknown function (DUF)21, a structure that is poorly characterised in terms of distribution among species and function [101, 102]. The C-terminal end of the protein also contains a CorC domain of unknown function. Expression of the archaeal *Methanoculleus thermophilus* CorB (MtCorB) and bacterium *Tepidiphilus thermophilus* (TtCorB) in liposomes showed transport of Mg^2+^ [101]. Expression of CorC in human embryonic kidney (HEK)293 cells showed Mg^2+^ extrusion when cells were exposed to Na^+^, which was prevented when Na^+^ was removed from the buffer. Mutational analysis of the *Thermus parvatiensi* CorC orthologue indicated that residue Asn94 (N94) might be important for Na^+^ sensitivity. Indeed, mutagenesis of this residue showed decreased efflux compared to wild type when overexpressed in HEK293 cells, being indicative that CorB/C might work as a Na^+^/Mg^2+^ antiporter [102].

The vertebrate orthologues of CorB were first identified in mouse, and named ancient conserved domain protein (ACDP) 1–4, which later were renamed as Cyclin M(CNNM)1–4 [103, 104]. Earlier studies suggested a nuclear function of the protein [103], more recent evidence indicates that it acts as a Mg^2+^ transporter in metazoa. Electrophysiological studies in *X. laevis* oocytes suggested that CNNM2 acts indeed as a Mg^2+^ exchanger [105]. Additionally, studies in HEK293 cells using the Mg^2+^ probe MagnesiumGreen demonstrated Mg^2+^ extrusion upon CNNM2 overexpression [106]. However, CNNM2 overexpression in HEK293 cells could only induce small Mg^2+^ and Zn^2+^-sensitive Na^+^ currents, which were ablated when a patient-derived CNNM2 mutant was used [107]. In addition, other groups could not repeat the results using the Mg^2+^-sensitive probe Mag-Fura-2 and failed to demonstrate changes in intracellular Mg^2+^ levels upon overexpression [108]. Moreover, transport studies with the stable isotope ^25^Mg^2+^ could not detect Na^+^-dependent Mg^2+^ transport in HEK293 cells [109]. Consequently, a Mg^2+^ sensor role for CNNM proteins was hypothesised. However, the IC_50_ value for MgATP binding of CNNM2 and CNNM4 are estimated to be approximately 160 and 45 μmol/L, respectively [110, 111]. It is, therefore, difficult to imagine that these proteins sense Mg^2+^ in the physiological situation.

The role of CNNM proteins remains disputed and has been discussed elaborately earlier [110, 111]. It should be mentioned that the different views may be dependent on the interpretation of findings using different in vitro models. Using magnesium sensitive probes, such as MagnesiumGreen and Mag-Fura-2, allows the determination of acute responses of CNNM protein upon various interventions, yet requires non-physiological concentrations of Mg^2+^. In contrast, studies using the stable isotope ^25^Mg^2+^ are superior in investigating Mg^2+^ fluxes, but do not provide information on intracellular Mg^2+^ concentrations. In addition, only overexpression studies have been performed that may not reflect the physiological situations. Recently, interaction partners have been identified that can modulate CNNM function. Cell-specific expression of these proteins could exert different effects based on the model used. For instance, phosphatase of regenerating liver (PRL)1–3 are proto-oncogenic proteins that can bind to CNNM proteins and are postulated to inhibit CNNM-mediated Mg^2+^ efflux [112–114]. Translation of the PRL proteins is induced upon a decrease in the intracellular Mg^2+^ levels [115], which could then inhibit CNNM-mediated efflux. The ADP-ribosylation factor-like proteins (ARL) 15 has recently been found to be involved in the glycosylation of the CNNM2 and CNNM3 and decreased CNNM3-mediated ^25^Mg^2+^ uptake upon overexpression [116]. A recent study has shown that CNNM proteins can interact with the Mg^2+^ channel transient receptor potential receptor type 7 (TRPM7) in vivo and vitro.[117] ARL15 reduced TRPM7-mediated currents in *X. laevis* oocytes upon heterologous overexpression, suggesting a potential, complex regulatory mechanism of ARL15-CNNM regulation on TRPM7 channel activity.

How the CNNM protein exert their function still remains to be determined, yet from a physiological point of view, it is clear for CNNM2 and CNNM4 that they are involved in systemic Mg^2+^ homeostasis. Patients suffering dominant mutations in the *CNNM2* gene present with hypomagnesaemia, seizures, and intellectual disability (HSMR) syndrome [107, 118, 119]. Patients have increased renal Mg^2+^ wasting, fitting with the expression of CNNM2 in the distal convoluted tubule within the nephron [107, 109, 118]. Systemic and kidney-specific knockout of *Cnnm2* in murine models and knock-down in zebrafish larvae have shown to result in mild hypomagnesaemia [120, 121]. Patients with recessive mutations in CNNM2 also suffer from brain abnormalities, such as demyelination and ventricular defects [118, 122]. *Cnnm2* knock out are embryonically lethal and may suffer exencephaly [121]. Similarly, *Cnnm4* knock mice also develop hypomagnesaemia, which is attributed to intestinal malabsorption [123, 124]. It is expressed at the basolateral membrane of colonic enterocytes and facilitates Mg^2+^ extrusion towards the blood compartment. Interestingly, patients suffering Jalili syndrome due to dominant mutations in the *CNNM4* gene do not develop serum Mg^2+^ disturbances, rather defects in amelogenesis and cone-rod dystrophy [125, 126].

Structurally, CNNM proteins are similar to CorB/C (Fig. 6B) [101, 102]. The CNNM orthologues contain multiple functional domains. At the N-terminus, a relatively long peptide sequence encodes for a signal peptide domain, that is cleaved off at the endoplasmatic reticulum and subsequently degraded [109, 119]. The proteins are then targeted to the plasma membrane after glycosylation which likely takes place in the Golgi-apparatus, mediated via ARL15 [116]. The transmembrane domain, the DUF21 domain, consists of three transmembrane spanning helices. A fourth domain, located between helices 1 and 2 was predicted to be a short re-entrant loop [109]. The structure for CorB/C proteins has recently been solved and has shown that this juxtamembrane domain forms a belt-like structure around the three transmembrane domains [101, 102]. The intracellular domain of CNNM proteins has extensively been studied. Similar to MgtE, CNNM proteins and CorB/C proteins contain CBS domains that bind MgATP [106, 127–129]. Binding of both, MgATP and free Mg^2+^ ions, results in conformational changes, rendering the protein in closed. The CNNM protein subsequently contains cyclic nucleotide bind homology (CNBH) domains which, in contrast with the initial predictions (so their name), do not bind cyclic nucleotides, and perhaps regulate the function of CNNMs [128, 130]. It has been proposed that this domain I) limits the conformational changes of the CBS domains upon binding of Mg^2+^/MgATP or II) functions as an adaptable regulator itself [128, 130]. One of the main differences with the CorB/C proteins is that the CNNMs have an extra transmembrane helix, that acts as a signal peptide and is cleaved off at the ER membrane, which is absent in CorB/C proteins (Fig. 6B) [109]. This results in a long N-terminal domain which is glycosylated and exposed to the extracellular space. In addition, studies have shown that the linker of the CBS1 and CBS2 domains is a target for binding of proteins such as PRL1-3 and ARL15 [112–114, 116]. Interaction partners of CorB/C proteins have yet to be identified, so it is not completely certain that these prokaryotic proteins are regulated in a similar fashion as the CNNM proteins. Furthermore, the CNBH domain has replaced the CorC domain in the CNNMs. Contrary to its name, it does not bind cyclic nucleotides, but is involved in dimerisation of the proteins and facilitating the conformational changes of the CBS domains, which (indirectly) affect Mg^2+^ efflux [128, 130]. The role of CorC domains in the CorB/C proteins in its function has not been studied thus far.

Apart from its homology to the CorB/C proteins, it is intriguing to mention a potential evolutionary link between MgtE and CNNMs as both proteins form dimers and contain CBS domains. It is interesting to speculate that MgtE split in two different proteins during evolution; into the Mg^2+^ transporting proteins SLC41 and the Mg^2+^ sensors CNNMs. However, as elaborately reviewed earlier[131], structural knowledge of the CBS domains gives us insights that do not support this theory. Although both proteins bind MgATP at the CBS domains, the structural consequences are quite different. First of all, the location of the ligand binding in the CBS domains is not conserved in the two proteins. While the motif where MgATP within the CBS domains binds is present in both proteins, MgATP binds at another site within the CBS domains in MgtE, unlike in CNNMs [85, 86]. This was discovered due to identification of mutations in CNNM4 (CNNM4-p.R407L) and CNNM2 (CNNM2-p.T568I) that cause the congenital disorders Jalili and HSMR syndrome, respectively [107, 118, 129]. Second, CNNM and MgtE proteins bind ATP and Mg^2+^ in a different manner [131]. Patch clamp studies in proteoliposomes indicated that the ability of intracellular Mg^2+^ to close the MgtE channel is dependent on the presence of ATP [85]. This suggests that presence of ATP determines Mg^2+^-sensitive gating of MgtE channels. Conversely, studies using a surface plasmon resonance sensorgram with immobilised, recombinant CBS domains from CNNM2 demonstrated binding of ionised Mg^2+^ to the CBS domains [106]. In contrast, ATP only binds in the presence of Mg^2+^. Taken together, these findings suggest that MgtE binds Mg^2+^ in an ATP-dependent manner, while conversely CNNMs bind ATP in a Mg^2+^ dependent fashion. Thirdly, only four Mg^2+^-bindings sites have been identified in CNNM orthologue, while MgtE contains seven. Lastly, binding of MgATP leads to “opening and closing” of the MgtE pore via the plug, while the CBS domains in CNNM adopt a “disc-like-flat” structure, which moves the DUF21 domain and closes the pore [85, 86, 101, 102, 114, 127, 130, 132]. Although these facts do not support MgtE as ancestor for the CNNM proteins, it cannot be ruled out that in time, MgtE orthologues adopted similar MgATP-binding properties as the current CNNMs.

### The eukaryotic Mg^2+^ channels TRPM6/7

The main entrance for Mg^2^ into metazoan cells is via the transient receptors potential melastatin type (TRPM) 6 and 7 channels, which are non-selective divalent cation channels, permeable for among others Zn^2+^, Mg^2+^, and Ca^2+^ [133, 134]. The protein subfamily TRPM is related to the TRP superfamily, consisting of cation transporters of which most respond to physical or chemical stimuli as reviewed extensively [88, 135–137].

TRPM7 is expressed ubiquitously in the body and considered the main gateway of Mg^2+^ into metazoan cells. Cells deficient of TRPM7 have decreased intracellular Mg^2+^ levels and require Mg^2+^ supplementation when cultured in vitro.[138, 139] The TRPM6 channel is more uniquely expressed with high levels in the intestines and kidney, contributing to Mg^2+^ homeostasis in vivo [133, 140, 141].

**Fig. 7:**
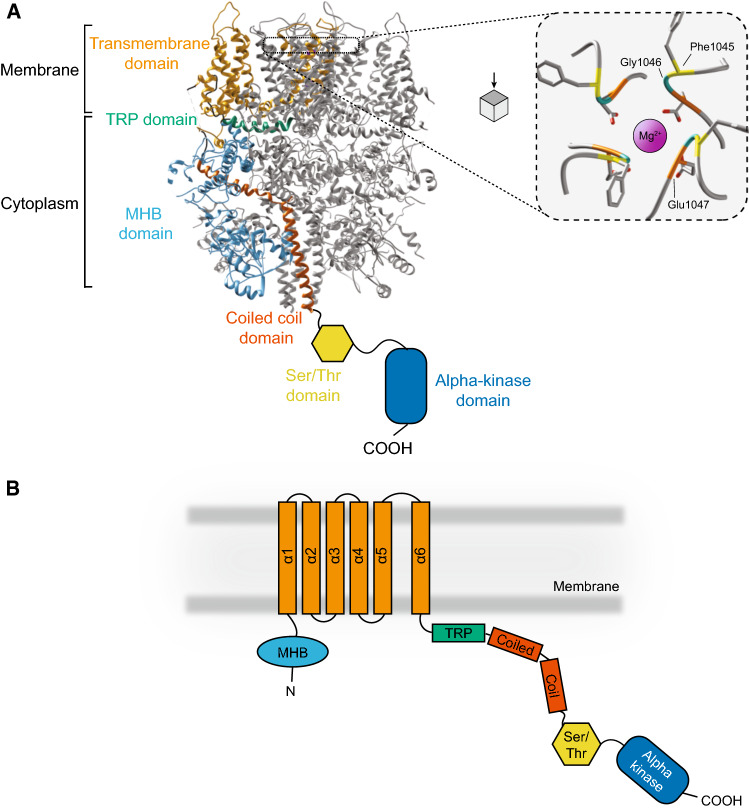
Structure of TRPM7; **A** Structure of the homotetramer transient receptor potential melastatin type 7 (TRPM7, PDB: 5ZX5) in complex with Mg^2+^ (purple spheres, left panel) with one monomer highlighted. Right panel: zoom in on residues of the selectivity pore Phe-Gly-Glu that bind Mg^2+^ **B** Schematic depiction of the monomer of TRPM7 using same colours as in A to reflect the approximate strucutre and location

No evidence has been found for prokaryotic TRP orthologues, yet different TRP subfamilies are found in unicellular eukaryotic organisms, such as algae (TRPP and TRPV), amoebozoans (TRPP and TRPML), and choanoflagellates, which are the closest single cell relatives to the metazoa (TRPML, TRPA, TRPV, TRPVL, TRPC, and TRPM), suggesting that the TRP channels evolved during the origin of the eukaryotes [142, 143]. Within the TPRM family, the members TRPM6 and TRPM7 stand out, as they are specialised in Mg^2+^ transport, yet structurally they are very similar to the other members of the TRPM channels (**Fig. ****7**). They share a conserved N-terminus, the melastatin homology region, that has been postulated to bind ligands and modulate channel activity, through a coiled coil region at the C-terminal domain of the protein [144–147]. Furthermore, the proteins all contain a TRP domain, a sequence of approximately 25 amino acids. This domain has been shown important in TRPV, -C, -M, and -L channels for their activity [148]. It binds to phosphatidylinositol (4,5) bisphosphate (PIP2), a common modulator of channel activity, although in some, it activates the channel, whereas in others causes, inhibition [135, 148]. The selectivity pore of TRPM7 is defined by its motif Phe-Gly-Glu (position 1045–47 in murine TRPM7) (Fig. 7A), facilitating Mg^2+^ and Ca^2+^ permeability. Mutagenesis of Glu1047 to Gln, which is found in this motif in TRPM4, a monovalent cation transporter, abolishes the divalent permeability [144].

TRPM6 and TRPM7 are specialised channels in the TPRM family for two major reasons. The channels form homo or heterotetramer structures, which is necessary for their activation. Yet, TRPM6 homotetramers are considered inactive, inferring that TRPM7 expression is always concomitant with TRPM6 [21, 134]. Secondly, both proteins are “chanzymes”, having a channel function and kinase domain. The structure of the kinase domain has not been solved yet, but from electrophysiological studies, the domain seems important for binding of ligands, such as MgATP, and is crucial for channel activity [149–152]. Deletion of the kinase domain leads to embryonic lethality in mice, while heterozygous deletion allows maturation of the animals, yet leads to defects in the hearth teeth and leads to decrease Mg^2+^ levels in the body [149, 153, 154]. In addition to Mg^2+^ and MgATP, Zn^2+^ and Ca^2+^ also directly regulate channel activity. Mg^2+^ is postulated to bind to the linker between the channel and kinase domain, whereas there is a Zn^2+^ binding motif located in the kinase domain, consisting of two histidines and cysteines [155]. Oxidation of the cysteines, for example induced by H_2_O_2_ exposure, results in dissociation of Zn^2+^ ions from the channel with inactivation as a consequence. These cysteines may, therefore, act as sensors for oxidative stress. Closing the TRPM7 channel may prevent further cell damage, as increased cytosolic Mg^2+^ levels are associated with increased ROS levels [156–158]. Furthermore, Zn^2+^ influx has been implicated with neurotoxicity, while increased intracellular levels may induce Ca^2+^-mediated caspase activity and ultimately cell death [159–161].

## Conclusions

Mg^2+^ homeostasis in both prokaryotes and eukaryotes is orchestrated by the interplay of various Mg^2+^ channels and transporters, indicating a high degree of regulatory pathways. Although the structure of individual Mg^2+^ transporters have significantly changed, the motifs that form the selective pore in CorA, CorB/C, and MgtE have all been conserved in their eukaryotic counterparts. Incredibly, the overall tertiary and quaternary structure for CorA and CorB/C have been sustained in MRS2 and CNNM proteins respectively despite low amino acid identity. This demonstrates the importance of these structures for Mg^2+^ specific transport.

Despite these similarities, it is conspicuous that in unicellular fungi, such as *S. cerevisiae*, CorA orthologues play an important role in Mg^2+^ homeostasis with many paralogues/homologues present in different subcellular compartments. However, metazoa only have the CorA orthologue, Mrs2, in mitochondria and do not have an evolutionary conserved CorA-like protein at the plasma membrane. Cellular Mg^2+^ influx is mainly orchestrated via TRPM6 and -7 channels in these organisms, suggesting that these channels may have an evolutionary advantage compared to CorA orthologues. TRPM6 and -7 are responsive to different hormones and ligands [138, 162, 163] which allows fine-tuning of their activity. Moreover, they contain a kinase domain of which the function of Mg^2+^ homeostasis is still poorly understood, despite extensive research. Unravelling the function of this domain, as well as further identification of interaction partners and regulatory pathways may shine light upon the loss of CorA orthologues in favour of TRPM6 and -7 channels in metazoa.

To gain more insights into the similarities and differences between pro- and eukaryotic Mg^2+^ channels/transporters, a few approaches could be considered. First of all, the structures of several eukaryotic Mg^2+^ channels and transporters have not completely resolved, including SLC41 and MgtE proteins. To date, only predication tools (AlphaFold) have provided structural information of SLC41 proteins, but ultimately cryo-EM or X-ray crystallography is required to elucidate the overall structures. Transport characteristics, e.g. permeation or gating dynamics, could be investigated if structures are available. This would especially be valuable for SLC41 proteins, because they lack the CBS domains compared to MgtE channels. Second, extensive phylogenetic tracing would allow to determine the evolutionary link of SLC41 and MgtE proteins, which is particularly interesting as MgtE orthologues appear to be missing in various phyla, such as land plants and fungi. In addition, it would be important to examine whether MgtA/B orthologues exist in eukaryotes. MgATPases have been postulated to be present in vertebrates, but have not been identified on a genetic level. Lastly, the mode of action of several transporters is often disputed, frequently due the use of different experimental techniques and models. Transport assays using specific isotopes or fluorescent probes in models such as liposomes directly would significantly contribute to the field. These assays are also valuable to determine the difference between paralogues, e.g. the CNNM or SLC41 proteins.

Of note, transporters discussed in this review may not be the only Mg^2+^ transporting proteins in eukaryotes. For instance, Magnesium transporter 1 (MagT1) was postulated as a Mg^2+^ transporter in X*. laevis* oocytes [164]. It is expressed at the plasma membrane and its expression is increased upon low-Mg^2+^ conditions in HEK293T cells [165]. Yet, mutations in the *MagT1* gene have been linked to N-glycosylation and immunodeficiency [166]. As many plasma membrane proteins are glycosylated, it is possible that MagT1 contributes to Mg^2+^ homeostasis by modulating Mg^2+^ channels and transporters at the membrane via glycosylation. Members of the solute carrier proteins 25 (SLC25) are mitochondrial specific antiporters of MgATP and HPO_4_^−^, that indeed transport (indirectly) Mg^2+^ [167]. Although members of these transporters can also transport ADP, free Mg^2+^ transport has not been observed. Lastly, the proteins Non-imprinted in Prader-Willi/Angelman syndrome (NIPA) 1–4 have been postulated to be Mg^2+^ transporters [19]. Mutations in the genes are linked to Prader-Willi/Angelman syndrome, resulting in hypogonadism, hypotonia, intellectual disability, growth defects, and childhood obesity [168]. Expression of NIPA protein in heterologous systems, such as the *X. laevis* oocytes, indeed show Mg^2+^ fluxes that can be ablated upon the introduction of patient mutations [169]. Yet, experimental data confirming the involvement of NIPA proteins in Mg^2+^ transport in mammalian cells is largely lacking. Extensive reviewing of aforementioned proteins should be performed in homologous cell systems to determine Mg^2+^ transport function.

In conclusion, to study the structural relationship between Mg^2+^ transporters in different phyla enables the understanding the origin and function of current mammalian magnesiotropic proteins. This broadens our current knowledge in Mg^2+^ homeostasis in health and disease.

## Data Availability

Not applicable.
